# Clinical Analysis and Applications of mRNA Vaccines in Infectious Diseases and Cancer Treatment

**DOI:** 10.7759/cureus.46354

**Published:** 2023-10-02

**Authors:** Paa Kwesi Ankrah, Ajibola Ilesanmi, Amos O Akinyemi, Victor Lasehinde, Oluwapelumi E Adurosakin, Oluwatobi H Ajayi

**Affiliations:** 1 Division of Infectious Diseases, Duke University, Durham, USA; 2 Center for Human Systems Immunology, Duke University, Durham, USA; 3 Department of Toxicology and Cancer Biology, University of Kentucky, Lexington, USA; 4 Department of Biology, Washington University in St. Louis, St. Louis, USA; 5 Department of Pure and Applied Biology, Ladoke Akintola University of Technology, Ogbomoso, NGA; 6 Division of Infectious Diseases, Duke Human Vaccine Institute, Duke University School of Medicine, Durham, USA

**Keywords:** mrna vaccine, hiv aids, aids, vaccinology, cancer, hiv, mrna, vaccine

## Abstract

Vaccination, for centuries, has been a potent preventive technique to treat morbidities. The messenger RNA (mRNA) vaccine technology is an innovative biomedical approach utilized in developing antigen-specific vaccines that can generate adaptive immune responses, triggering both humoral and cellular immunity to enhance the body's defense against specific infections. This review provides a comprehensive, comparative analysis of mRNA vaccine technology and conventional vaccines by focusing on the structures, components, and classifications. An exploratory analysis of the similarities and differences between mRNA vaccine technology and live-attenuated vaccines highlights the mechanisms by which mRNA vaccines elicit immune responses. This review extensively discusses the production, stability, synthesis, and delivery processes associated with mRNA vaccines, showcasing the advancements and technological superiority of this approach over conventional vaccine technologies. Additionally, the potential of mRNA vaccine technology as a potent alternative for the development of vaccine candidates targeting HIV and cancer is examined.

## Introduction and background

Vaccination is a globally recognized effective approach to combating infectious and malignant diseases [1], serving as the most cost-effective method for preventing infections [2]. As a result, significant ongoing studies focus on the development of novel vaccines. With the global population on the constant rise and the emergence of new pathogens, vaccinology has become an essential field within clinical science, public health, and biomedical research [3]. Vaccines have proven their efficacy in providing protection against diseases like smallpox, measles, and poliomyelitis, leading to the eradication of some of these illnesses [4]. These successful vaccine administrations lay the foundation for advancing vaccination techniques that align with current epidemiological needs.

The administration and mechanism of vaccines can be a complex process for certain diseases, such as the influenza virus, due to the variability of the pathogen and its ability to evade host immunity even in vaccinated populations [5]. In such cases, the effectiveness of vaccines often depends on the vaccine type and the pathogenic strain. Additional drawbacks, such as waning immunity and varying immune mechanisms, for example, whooping cough vaccines [6], raised concerns about the development of multifunctional vaccines. In challenging conditions like HIV, there is a pressing need for vaccines that can rapidly elicit a functional response and overcome the difficulties associated with strain variation [7, 8]. Therefore, the development of new vaccines should be grounded in a practical understanding of the interactions between pathogens and hosts at the cellular and molecular levels [9].

In order to achieve enhanced vaccine efficacy, nucleic acid vaccines, specifically messenger RNA (mRNA) vaccines, have emerged as promising prospects in the field of vaccinology, primarily due to their ability to elicit both humoral and cellular immunity [10]. This article performs a comprehensive exploratory analysis of mRNA vaccine technology and conventional vaccines. Conventional vaccines, although successful in the past, do not efficiently address the rising global health challenges. The focus is on understanding the immune response mechanism, the synthesis of mRNA-based vaccines, and the effective clinical translation of this technology. Additionally, the potential use of mRNA to develop vaccines against HIV/AIDS, aging-associated morbidities such as cancer, and future therapeutic advancements is explored.

## Review

Overview of mRNA vaccine technology

Although the knowledge surrounding the therapeutic properties of mRNA has spanned about three decades [11], the technology has recently been the prime focus in vaccinology. This is primarily due to current possibilities regarding the efficacy of these nucleic acids and how they can be used in disease prevention. Originating from their use in protein production [12] and their ability to elicit an immune response in mice [13], recent advancements have revealed the greater potential of mRNA vaccines. Documentations indicate the progression from preclinical data generation to human clinical trials. [10].

**Figure 1:**
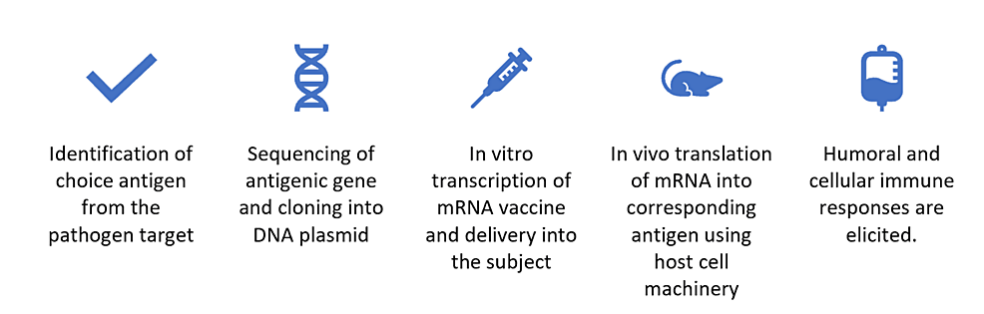
Workflow of mRNA vaccinology. Summary of the sequential procedures in mRNA vaccinology, sequence identification (far left) through in vivo clinical trials to stimulate the appropriate humoral and cellular responses (far right).

During the COVID-19 pandemic which demanded the rapid development of vaccines to forestall the number of deaths and minimize the effect of the virus, different strategies, such as the use of DNA vaccines, viral vectors, mRNA, and protein subunits, were employed to develop vaccines [14]. Among the approaches employed, mRNA vaccines have been successful in combating the pandemic [15]. The rapid production of mRNA vaccines represented a significant advantage, especially in the face of a highly mutating pathogen, which reinforced the importance of vaccines as major elements in combating emerging pathogens. The mRNA vaccine technology is an advanced technique built upon the principles of live-attenuated vaccines and subunit vaccines [16]. They offer the advantage of being safer and more antigen-specific compared to live-attenuated vaccines and do not require the administration of adjuvants [17]. Through the transfer of RNA transcripts encoding immunogens into the cells, translation results in the generation of proteins in a manner similar to a viral infection but with the capacity to improve the body's immunity [18]. An illustrative workflow of mRNA vaccinology is shown in Figure 1.

Structural composition of mRNA vaccines

The mRNA vaccines are composed of a cap structure made of 7-methylguanosine connected to a nucleotide, a tail of 40-120 adenosine residues known as the poly (A) tail, and the gene of interest, which is flanked by a 5' and 3' untranslated region [17]. The components of the mRNA vaccines are essential for the efficiency of the technology. While the cap structure effectively blocks recognition by the RNA sensor in the host's cytoplasm, the length and structure of the untranslated regions are vital in promoting translation to facilitate gene expression [19, 20]. To protect the mRNA from degradation, the length of the poly (A) tail is a crucial factor [21]. Specific nucleoside modifications and sequence engineering can be carried out to improve translation efficiency [22]. The mRNA vaccines are broadly categorized into non-replicating and self-replicating, each capable of eliciting an adaptive immune response [18]. The distinction lies in the presence of RNA-dependent RNA polymerase, which is a complex derived from the genome of a positive-sense single-stranded RNA virus and is exclusive to self-replicating mRNA vaccines [23]. This complex plays a crucial role in the self-amplification process specific to self-replicating mRNAs. Consequently, these two classes exhibit several differences. Non-replicating mRNA constructs are small, uncomplicated, and lack additional encoded proteins, thereby minimizing the risk of unintended immune responses [24]. This characteristic contributes to their enhanced stability, albeit with shorter half-lives.

Self-replicating mRNAs are characterized by their larger size, attributed to the presence of the self-amplification complex [23]. While this larger size offers advantages in terms of sustained expression and increased immunogen production, it poses a significant challenge to their stability [25]. Attempts to optimize self-replicating mRNAs, such as nucleoside modification, are constrained due to the potential impact on their self-amplifying capacity [26]. Multiple predominant variables that differentiate mRNA vaccines from conventional vaccines are listed in Table 1.

**Table 1:** Logistics and comparative analysis of mRNA vaccines and conventional vaccines

Factor	Conventional Vaccines	mRNA Vaccines	References
Host cell safety	Conventional vaccines, like live-attenuated vaccines, may not be the optimal choice for generating vaccines targeting highly pathogenic antigens such as poliovirus type 1, primarily due to the reversion risk involved.	When mRNA vaccines are used to produce these virulent strains, there is no associated risk of integrating into the host genome. Therefore, it cannot undergo the risk of reversion. Also, since the activity of mRNA is temporary, it can be effectively decomposed in the host without resulting in future complications.	[27]
Synthesis	Conventional vaccines take longer to produce as they sometimes require whole-pathogen cultivation and propagation, followed by a continuous need for serial passaging to ensure safety.	Production of mRNA vaccines, specifically self-amplifying mRNA, can occur in about a few hours through a cell-free enzymatic transcription reaction, and in vitro transcription can be utilized. Also, due to the similarities in their production and purification processes, new mRNA vaccines can be easily produced once the genomic sequence of a target antigen has been identified.	[3, 28]
Scalability	Production involves complex purification processes which are usually expensive	The mRNA vaccines are cell-free and can be easily scaled up, hence, less expensive to produce.	[29]
Pliancy	Most conventional vaccines are not readily amenable to modifications.	The mRNA vaccines can be easily modified to eliminate undesired effects or enhance responses to different antigenic strains of the pathogens.	[26]
Reproducibility	There are existing concerns about the reproducibility of virus cultures and the production of proteins in mammalian cells.	They are highly reproducible.	[28]
Route of administration	Prior vaccines were mostly DNA-based (for example, influenza and hepatitis C vaccines) and required direct delivery into the host nucleus.	The mRNA vaccines are administered into the cytoplasm and do not enter the nucleus.	[28]
Adjuvant administration	In conventional vaccine administration, there is a need for the introduction of adjuvants to stimulate a robust immune response.	Adjuvants are not required to administer mRNA vaccines as they can induce a strong immune response.	[30, 31]
Response to emerging diseases	Due to the longer duration required to produce conventional vaccines, they are unsuitable for rapid response measures to emerging infections.	The mRNA vaccines can be rapidly produced in response to emerging infections or pandemics.	[29]
Antigen-specificity	Antigens used in producing these vaccines are specific to the infection under consideration, so conventional vaccines are mostly antigen-specific.	The mRNA vaccines may contain a mix of multiple sequences, which would provide broad coverage to express all kinds of proteins that would also meet specific genetic requirements.	[32]
Immune response	Most live attenuated vaccines and subunit vaccines are not capable of eliciting CD8 T cell responses in humans.	The mRNA vaccines can elicit CD8 T cell responses, which are beneficial in eradicating infections and can potentially eradicate tumors.	[32]

From a clinical perspective, mRNA vaccines can be swiftly designed and manufactured within a matter of weeks, allowing for easy modifications to target multiple variants. This flexibility provides a significant advantage in addressing emerging diseases and coping with diverse antigenic strains. Compared to live-attenuated and killed vaccines, mRNA vaccines have emerged as a primary focus in vaccine production due to their straightforward design and synthesis [33], long-term stability during storage [34], and remarkable effectiveness in triggering both cellular and humoral immune responses [12]. The mRNA used for vaccine purposes can be generated in vitro using cell-free systems or extracted from specific target cells. In cell-free systems, the synthesis of vaccine-targeted mRNA necessitates a complete DNA template encoding functional mRNA, including ribonucleotides, polymerase, and a synthetic 5' cap analog [35]. While mRNA vaccines offer significant advantages, they also encounter certain challenges.

Notable drawbacks include degradation by ubiquitous ribonucleases, which necessitate precise delivery into the cytoplasm of target cells (in vivo) and enhancing inherent adjuvant functions. Naked extracellular mRNA can be taken up by various cells through micropinocytosis and caveolae mechanisms. However, the efficiency of this internalization process is often insufficient, requiring the packaging of mRNA within amphipathic vectors to mask its large negative charge and hydrophilicity. These amphipathic vectors typically consist of lipid-based, polymer-based, or hybrid materials. The optimal mRNA delivery system should provide protection against ribonucleases, facilitate efficient cell entry and escape from endosomes, and effectively target lymphoid organs [36].

Synthesis and stability

The mRNA vaccines are synthesized based on the eukaryotic mRNA blueprint: a 5' cap, 5' and 3' untranslated regions (UTR), and a 3' poly(A) tail [37]. The synthetic 5' cap, designed to resemble the wild-type eukaryotic mRNA, interacts with the cap-binding factor eIF4E to recruit the 43S pre-initiation complex [38]. The addition of synthetic cap analogs, such as m7GpppG, to the in vitro transcription process easily accomplishes capping [39]. These 5' cap analogs are incorporated in both the forward and reverse directions, although only the caps added in the forward direction remain functional due to the use of anti-reverse cap analogs (ARCA), such as 7-methyl (3'-o-methyl) GpppG [10, 40]. Another 5' capping approach involves introducing recombinant capping enzymes derived from the vaccinia virus after in vitro transcription, ensuring sufficient capping of the 5' ends with increased resistance to decapping pyrophosphatases [41]. Further mRNA translation necessitates the incorporation of a poly(A) tail through two methods. First, by transcribing a DNA template with a predetermined poly(A) length. Second, by employing enzyme-mediated polyadenylation to generate mRNAs with varying poly(A) lengths [42, 43]. Moreover, optimizing mRNA stability can be achieved through subtle modifications such as the addition of 5' and 3' untranslated regions [44]. The replacement of rare codons with synonymous codons can enhance translation efficiency [45], and base-specific modifications can protect mRNA from degradation by ribonucleases (RNases). It is noteworthy that the creation of site-specific single nucleotide polymorphisms in the poly(A) tail, other than adenine, can significantly decline the overall expression of the mRNA. Several studies have explored the development of self-amplifying replicons by integrating viral replicase coding sequences into the mRNA aimed at stimulating immunogenicity [45, 46].

Delivery mechanisms of mRNA vaccines

Various vectors, including lipid-modified, polymer-based, or hybrid systems, are used to shuttle mRNA vaccines into cells. Two main lipid-modified vectors are lipoplexes, a combination of liposomes and nucleic acids, as well as lipid nanoparticles (LNPs). Liposomes offer the advantages of low toxicity, easy synthesis, and efficient biodegradability [47]. The interaction between cationic liposomes and mRNA leads to the formation of lipoplexes. The lipids used in lipoplex construction for mRNA vaccine delivery can be cationic, such as 1,2-dioleoyl-3-trimethylammonium-propane (DOTAP), or zwitterionic, such as 1,2-dioleoyl-sn-glycero-3-phosphoethanolamine [48]. However, a major limitation is the rapid clearance of cationic lipids, enforcing the use of alternatives like ionizable lipids. Ionizable lipids, employed as part of LNPs, offer higher transfection efficacy by remaining neutral at physiological pH. The LNPs typically consist of polyethylene glycol (PEG), ionizable lipids (or a cationic lipid), helper phospholipids, and cholesterol. The ionizable lipids undergo ionization as the pH decreases, ensuring endosomal escape by maintaining a neutral or slightly cationic charge. Cholesterol and helper phospholipids are incorporated to ensure particle stability and maintain the lipid bilayer structure, respectively. Polyethylene glycol reduces non-specific interactions with plasma proteins, thereby extending circulation time [49]. Reports have demonstrated the increased effectiveness of LNPs in delivering small interfering RNA (siRNA) [50-52] and mRNA vaccines against the Zika virus [53, 54].

A comparison of polyplexes and lipoplexes demonstrated the higher overall stability of the molecules in polyplexes [55]. Polymer-based vectors offer the advantage of easy optimization of molecular size, ligand-binding sites, and geometry (linear or branched) [56]. One frequently used type of polyplex nanoparticle is cationic polyethyleneimine (PEI) [57]. Self-amplifying mRNA nanoparticles synthesized with PEI (22kDa) or histidylated PEI (34.5kDa) have shown improved efficiency in delivering mRNA vaccines to dendritic cells, eliciting both cellular and humoral immune responses [58]. Another type of polymer-based vector is the micelleplex, which involves the formation of micelles using amphipathic co-polymers. Notably, polyplexes do not form micelles [58]. Micelleplexes have been explored for non-vaccine administration of mRNA in areas such as protein replacement [59], cancer therapy [60], and cellular reprogramming [61]. Hybrid vectors, including lipopolyplexes and cationic nano-emulsions, consist of a combination of different materials. Lipo-polyplexes provide excellent protection against mRNA degradation and offer the advantages of both polyplexes (higher transfection rates, escape from endosomes, and improved stability) and lipoplexes (enhanced cellular uptake and minimal cytotoxicity) [62].

Induction of host immune response by mRNA vaccine

The generation of innate and adaptive immune responses plays a crucial role in the efficacy of vaccines, and this principle is observed in the mechanism of action of mRNA vaccines. Similar to live-attenuated vaccines, mRNA vaccines trigger localized inflammation at the injection site to recruit antigen-presenting cells and facilitate pathogen neutralization through antibody production [63]. To achieve this, the vaccine utilizes pattern recognition receptors to detect pathogens, establishing a connection between the innate and adaptive immune systems and enhancing the immunological response against the pathogen. When non-immune cells internalize mRNA vaccines at the injection site, the expression and cellular localization of vaccine components depend on the specific site of injection. Moreover, the efficacy and safety of the vaccination are largely influenced by the chosen route of vaccine administration [63].

**Figure 2:**
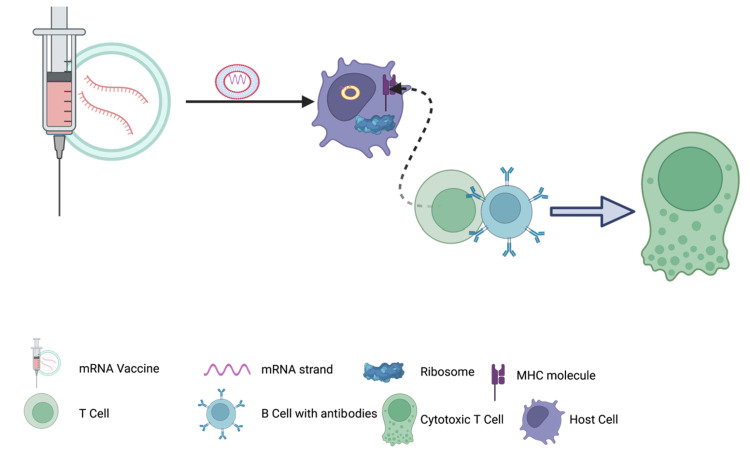
A schematic diagram of how the mRNA vaccine induces the host immune response. The mRNA, encapsulated within a lipid nanoparticle, is phagocytosed by dendritic cells for further translation into antigen proteins. These antigens are presented on the dendritic cell's surface using MHC molecules, resulting in the activation of T cells and B cells. Ultimately, this coordinated process generates humoral immunity and cytotoxic cell response. MHC: major histocompatibility complex

The administration of naked mRNA through intramuscular injection leads to its expression in myocytes, fibroblasts, and keratinocytes. Alternatively, intradermal administration causes mRNA expression mainly in the dermis [64]. The precise mechanism by which mRNA is delivered to the cytoplasm remains elusive, but it is hypothesized to occur either through endosomal uptake or the involvement of low-density lipoproteins expressed in most cells [28]. Upon expression of the mRNA molecule in the cells, chemokines and cytokines are upregulated due to the sensory characteristics of toll-like receptors and specific genes like the retinoic acid-inducible gene (RIG). These pattern recognition receptors stimulate an increase in tumor necrosis factor (TNF) to accelerate the expression of matrix metalloproteinases. Metalloproteinases break down neighboring tissues while guiding dendritic cells toward the lymph nodes. Consequently, mRNA can be detected in the lymph nodes and surrounding tissues, leading to the proliferation of B cells and granulocytes. The expression of the vaccine antigen in these cells triggers the production of antigen-specific antibodies and CD8+ T cell responses. The mRNA present in the lymph nodes undergoes translation into polypeptides, which are subsequently presented to major histocompatibility complex (MHC) molecules on the cell surface. The schematic mechanism of immune response elicitation by mRNA vaccines is shown in Figure 2.

Role of dendritic cells (DCs) in mRNA vaccinology

Dendritic cells (DCs) are highly receptive to mRNA transfection and play a crucial role in triggering immune responses. They efficiently deliver entire antigens to B cells, promoting an antibody-mediated reaction. Loading full-length tumor antigens onto dendritic cells has the potential to enhance immunotherapy by inducing broad T-cell responses, irrespective of the patient's human leukocyte antigen (HLA) type [65]. In this approach, tumor-associated antigens (TAAs) are loaded onto DCs in the form of defined peptides. By exposing DCs to the complete length of the TAA, a diverse range of epitopes from the TAA can be presented by the patient's unique set of HLA molecules. This technique stimulates a broader repository of T cells compared to peptide-antigen-loaded DCs, generating T cells specific to known and unidentified TAA epitopes presented by different HLA types. Generic stimulation of T cells potentially benefits larger patient populations, making DC therapy an effective procedure for mRNA transfection, either in vivo or ex vivo [66].

Messenger RNA (mRNA) technology in HIV vaccine development

The success of mRNA vaccines against SARS-CoV-2 has sparked the interest of researchers in exploring the potential of mRNA vaccine technology for developing an HIV vaccine. Despite decades of intensive research, HIV remains a significant global public health challenge, with 39 million people living with HIV and 630,000 recorded deaths in 2022 [67]. Currently, antiretroviral therapy (ART) and pre-exposure prophylaxis (PrEP) are the primary strategies for HIV prevention and treatment. However, the high cost and limited accessibility of these medications in developing countries and low-income nations contribute to the increasing number of cases. Unfortunately, significant progress in HIV vaccine development has been elusive, with only the RV 144 clinical trials demonstrating approximately 31% efficacy [68].

**Figure 3:**
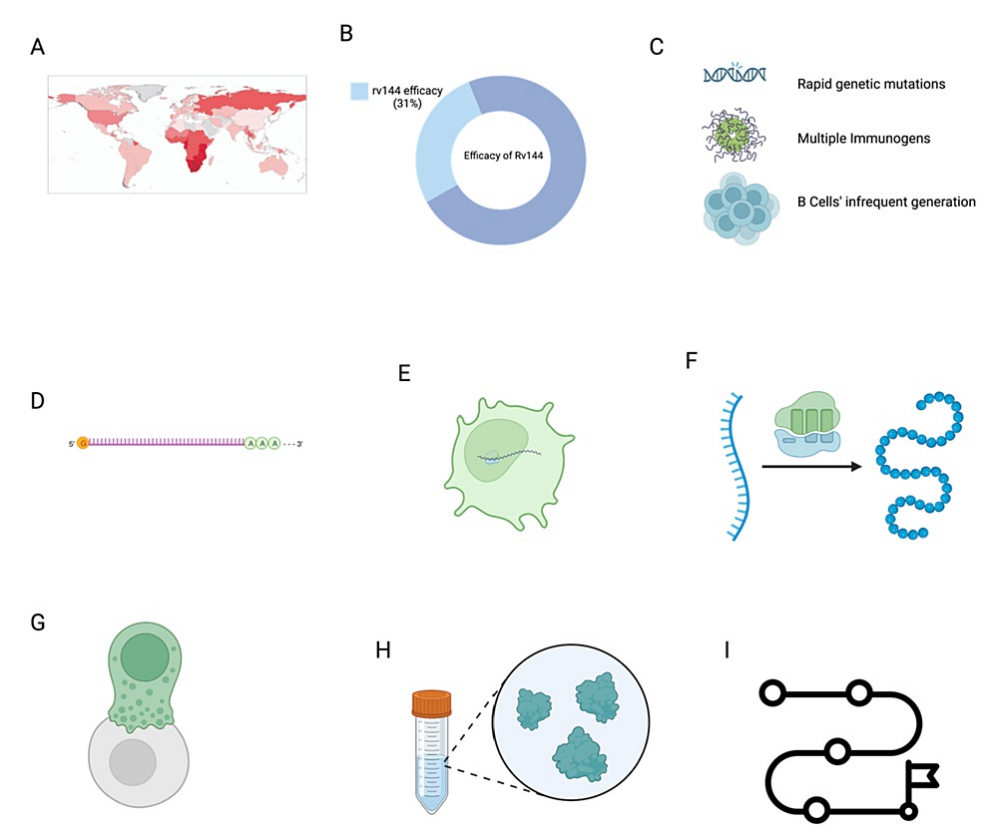
(A) Global HIV statistics: a global heatmap of HIV, highlighting the most affected regions; (B) The RV_144 trial demonstrated 31% efficacy in clinical trials, underscoring the limited success of previous HIV vaccine efforts; (C) Barriers to traditional HIV vaccine development, such as rapid genetic mutations and challenges in eliciting appropriate immune responses; (D) Structure of the encapsulated mRNA forming the backbone of the vaccine; (E) Cellular uptake: mRNA entry and incorporation into the host cellular machinery to ensure vaccine viability; (F) Protein translation to create viral proteins that induce an immune response; (G) Immune response activation: production of broadly neutralizing antibodies (bnAb) and activation of CD8+ T cells. These are two key determinants of a vaccine's efficacy; (H) Representations of current HIV prevention strategies like ART and PrEP (I) A roadmap delineating the stages of mRNA-based HIV vaccine development from laboratory research through clinical trials to potential global implementation ART: antiretroviral therapy; PrEP: pre-exposure prophylaxis [70]

The ideal HIV vaccine would stimulate both cell-mediated immunity and humoral immunity against HIV-1 and HIV-2. Moreover, the primary host defense mechanism involves the production of antibodies that can neutralize the virus and prevent infection in the host. For viruses that can evade neutralizing antibodies (bnAb), a secondary line of defense is necessary, which involves the activation of cytotoxic CD8+ T cells [69]. These T cells combat such viruses and have been identified as a potential vaccine for HIV 2. Developing an effective HIV vaccine that can elicit HIV-neutralizing antibodies (bnAb) has been a significant challenge [70], particularly with traditional vaccine development strategies. There are several roadblocks to this approach, including the high level of genetic mutations required in germinal centers to generate bnAbs, the need for multiple immunogens for sequential immunizations to induce bnAbs, and the infrequent generation of B cells that can bind to HIV-neutralizing epitopes, leading to immune tolerance [71]. The mRNA vaccine provides an alternative method to seamlessly administer vaccines through micelleplexes, polyplexes, and lipo-polyplexes into targets within the host. As represented in Figure 3, recent advances in mRNA vaccine formulation and their application in HIV vaccination have provided opportunities for further exploration in this field.

The mRNA cancer vaccine 

Cancer mRNA vaccines offer a practical prognosis for cancer therapy, combining specificity, safety, and the potential for a long-term immunotherapeutic response through immunologic memory. The viability of mRNA vaccines was demonstrated in 1990 when direct injection of in vitro transcribed (IVT) mRNA into mouse skeletal muscle cells resulted in effective mRNA expression [72]. Faghfuri and associates categorized cancer vaccines into different types, including nucleic acid-based (DNA/RNA) vaccines, tumor- or immune-cell-based vaccines, peptide-based vaccines, and viral vector-based vaccines [73]. They further hypothesized that DNA- or RNA-based vaccines, similar to other vaccines, are safe and well-tolerated. These vaccines are non-infectious and produced without protein or virus contamination, making them suitable for both prophylactic and therapeutic applications [74]. Clinical studies have predominantly focused on mRNA as the basis for RNA-based vaccines. These mRNA cancer vaccine platforms surpass traditional vaccination with respect to efficacy, safe administration, rapid development potential, and cost-effective production. Naked mRNA vaccines, when exposed to antigen-presenting cells (APCs), effectively induce the expression of tumor antigens. The process activates APCs and stimulates the innate and adaptive immune systems [75]. The underlying concept of using mRNA as a cancer vaccine platform revolves around delivering desired transcripts encoding tumor-associated antigens (TAAs) or tumor-specific antigens (TSAs) into the cytoplasm of host cells, typically APCs [38]. The mRNA vaccination has the capacity to elicit responses from CD4+ T cells, CD8+ cytotoxic T lymphocytes, as well as humoral responses mediated by antibodies and B cells. These immune responses collectively contribute to the effective elimination of cancerous cells [76].

The current research focuses on cancer vaccines, including non-replicating modified mRNA, modified mRNA, and virus-derived self-amplifying mRNAs (SAM). Detailed comparisons among these three mRNA types have been extensively reviewed [77, 78]. Since its initial discovery in 1990, in vitro transcription (IVT) has been widely utilized to synthesize both modified and unmodified non-replicating mRNA as well as SAMs. This technique involves employing a linearized DNA template containing the target antigen sequences and a bacteriophage RNA polymerase, such as T3, T7, or SP6 RNA polymerase [79]. Additionally, mRNA is modified to resemble fully mature mRNA molecules naturally present in the cytoplasm of eukaryotic cells. Once the mRNA or SAM is internalized and transported to the cytosol, ribosomes read the mRNA, translate it into proteins, and subsequently post-translationally modify those proteins to generate correctly assembled functional proteins. This process ensures the production of functional proteins that can contribute to the desired immune response. The delivery method of mRNA vaccines cannot be overemphasized. To achieve therapeutic relevance, efficient in vivo and ex vivo distribution of mRNA is essential. Currently, two primary methods for administering mRNA vaccines have been identified. The first method involves loading mRNA into dendritic cells ex vivo, followed by re-infusion of the transfected cells. The second method entails the direct injection of mRNA into the parenteral space, with or without a carrier [79]. Both methods have been extensively explored using various strategies, including naked mRNA, cationic nano-emulsion, electroporation, modified dendrimer nanoparticle, protamine liposome, protamine, cationic polymer, cationic polymer liposome, polysaccharide particle, cationic lipid cholesterol (polyethylene glycol) PEG nanoparticle, cationic lipid cholesterol nanoparticle, and cationic lipid nanoparticle [78]. The investigation of these approaches aims to optimize mRNA vaccine delivery and enhance their efficacy.

## Conclusions

The development of therapeutic mRNA vaccine candidates is a complex process with meticulous procedures. The emergence of mRNA vaccine technology, which combines the principles of live-attenuated vaccines and subunit vaccines, represents a significant step in the right direction. Additionally, the rapid, safe, and specific production of mRNA vaccines offers a substantial advantage in effectively combating both known and newly discovered pathogens, making vaccination efforts more resourceful. Although mRNA vaccine technology holds promise for the development of vaccine candidates against diseases like cancer and the HIV virus, it is noteworthy that further biomedical research is necessary. This article highlights progress in mRNA vaccinology, introduces the current limitations, and provides a recent knowledge base for further research.
